# Recurrent Loculated Pleural Effusions Due to Pleuropulmonary *Paragonimus westermani* Infection

**DOI:** 10.4269/ajtmh.23-0301

**Published:** 2023-08-07

**Authors:** Shihab Sarwar, Alexander J. Kipp, Stephen D. Vaughan

**Affiliations:** ^1^ Department of Medicine, University of Calgary, Calgary, Canada;; ^2^ Division of Microbiology, Department of Pathology and Laboratory Medicine, University of Calgary, Calgary, Canada;; ^3^ Division of Infectious Diseases, Department of Medicine, University of Calgary, South Health Campus, Calgary, Canada

**Figure 1.**
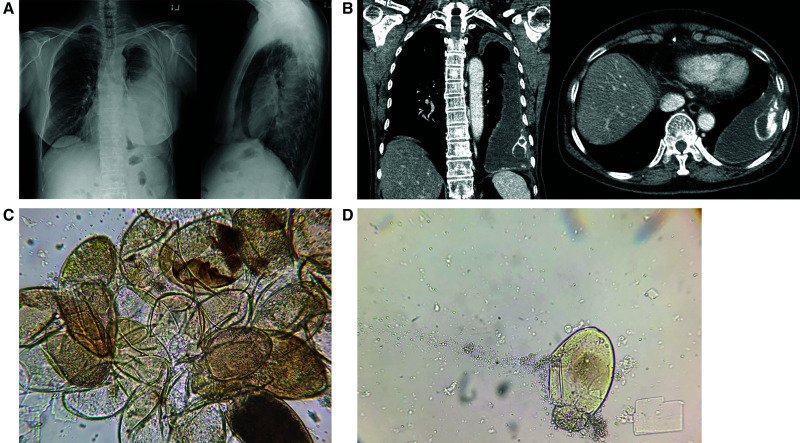
(**A**) Chest x-ray posteroanterior and lateral views demonstrating large loculated left-sided pleural effusion. (**B**) Chest computed tomography, contrast enhanced in coronal and axial cuts, showing loculated left pleural effusion with curvilinear ossification inferiorly. (**C**) Wet mount images derived from pleural fluid specimen demonstrating multiple *Paragonimus westermani* ova clumped together. (**D**) A single *P. westermani* ovum.

A 72-year-old South Korean woman presented with chronic progressive dyspnea and large loculated left-sided pleural effusion (Figure 1A). Her complete blood count was unremarkable with no eosinophilia. Thoracentesis revealed exudative yellow turbid fluid with pH 7.09 (normal: > 7.30), glucose < 0.3 mmol/L (normal: 3.3–11 mmol/L), white blood count 740 × 10^6^/L (no differential), and lactate dehydrogenase (LDH) 1,705 U/L with serum LDH 178 U/L (normal: 100–235 U/L). Bacterial (aerobic and anaerobic) and fungal cultures of the pleural fluid were negative. Microscopic examination of the fluid showed ova of *Paragonimus westermani* (Figure 1C and D). Ten years prior, she was diagnosed with pulmonary *P. westermani* infection by microscopic *Paragonimus* ova on thoracentesis and treated with oral (PO) praziquantel 600 mg three times daily (TID) × 3 days. She acknowledged remote pulmonary *P. westermani* infection 40 years prior for which she was treated in South Korea. She denied ingestion of raw crustaceans or seafood and had last traveled to South Korea 15 years prior. She was treated with praziquantel 1,500 mg (25 mg/kg) PO TID for 2 days and was discharged. *Paragonimus* spp. ELISA serology was positive with an optical density of 2.9 (positive if > 1.5). At follow-up, there was re-accumulation of the left pleural effusion requiring pleural drainage. She was then treated with triclabendazole and continued to have recurrent pleural effusions that were managed conservatively.

*Paragonimus westermani* is a foodborne infection transmitted through ingestion of raw crustaceans and is endemic to multiple Asian, African, and South American countries.^1^ Paragonimiasis is treated with praziquantel, and alternative treatment is with triclabendazole.^2^ There are reports of recurrent pleural effusions caused by pulmonary paragonimiasis treated with pleural drainage and four cycles of praziquantel at 75 mg/kg for 2- to 3-day courses at 3-week intervals with cure.^3^^,^^4^ Expected duration of ova persistence in pleural fluid after treatment and fluid evacuation is unknown. Treatment is effective only against the adult fluke, not the ova.

This case demonstrates a rare scenario of recurrent pleural effusion from *P. westermani* infection. It is unlikely that this was a result of repeated infections because of lack of travel and dietary history. The adult *Paragonimus* spp. life span is up to 20 years; however, given her recurrent presentations over 10 years (confirmed in Canada) and 40 years (including effusion drained) in Korea plus her directly observed completion of first- and second-line treatments make it unlikely she still had live *Paragonimus* adult flukes excreting ova. Most likely, this represents chronic pleural inflammation related to the large ova burden released from a chronic calcified sequestrum with ossification (Figure 1B) rather than inadequate treatment or antiparasitic resistance.
